# Night shift work and risk of breast cancer in women: the Generations Study cohort

**DOI:** 10.1038/s41416-019-0485-7

**Published:** 2019-05-29

**Authors:** Michael E. Jones, Minouk J. Schoemaker, Emily C. McFadden, Lauren B. Wright, Louise E. Johns, Anthony J. Swerdlow

**Affiliations:** 1 Division of Genetics and Epidemiology, The Institute of Cancer Research, London, SW7 3RP UK; 2 Division of Breast Cancer Research, The Institute of Cancer Research, London, SW7 3RP UK; 3 Present Address: Nuffield Department of Primary Care Health Sciences, University of Oxford, Oxford, OX2 6GG UK

**Keywords:** Risk factors, Breast cancer, Cancer epidemiology

## Abstract

**Background:**

It is plausible that night shift work could affect breast cancer risk, possibly by melatonin suppression or circadian clock disruption, but epidemiological evidence is inconclusive.

**Methods:**

Using serial questionnaires from the Generations Study cohort, we estimated hazard ratios (HR) and 95% confidence intervals (95%CI) for breast cancer in relation to being a night shift worker within the last 10 years, adjusted for potential confounders.

**Results:**

Among 102,869 women recruited in 2003–2014, median follow-up 9.5 years, 2059 developed invasive breast cancer. The HR in relation to night shift work was 1.00 (95%CI: 0.86–1.15). There was a significant trend with average hours of night work per week (*P* = 0.035), but no significantly raised risks for hours worked per night, nights worked per week, average hours worked per week, cumulative years of employment, cumulative hours, time since cessation, type of occupation, age starting night shift work, or age starting in relation to first pregnancy.

**Conclusions:**

The lack of overall association, and no association with all but one measure of dose, duration, and intensity in our data, does not support an increased risk of breast cancer from night shift work in women.

## Background

Over 30 years ago, it was proposed that suppression of the pineal hormone melatonin by exposure to electric light at night could increase risk of breast cancer.^1^ In 2007, an International Agency for Research on Cancer working group concluded that there was limited evidence in humans for the carcinogenicity of shift work that involves night work, but overall shift work that involves circadian disruption is probably carcinogenic to humans (Group 2A).^2^ There are biological reasons why night shift work may increase risk of breast cancer by suppression of melatonin (the ‘melatonin hypothesis’)^3^ or disruption of internal ‘body clocks’ (circadian clocks),^4^ however, findings from epidemiological cohort^5–16^ and case-control^17–22^ studies have been inconclusive. Most,^17–21,23–25^ but not all,^15^ meta-analyses suggest night shift work may be associated with a modestly raised risk of breast cancer in women.^26,27^ But the association is weaker when limited to cohort studies,^18–21,25,27^ the type of night shift work and exposure definitions have varied from study to study,^28^ and no clear dose–response relationship has been demonstrated.^26,27^

The most recent meta-analysis of all prospective studies, including three previously unpublished cohorts concluded that night shift work has little or no effect on breast cancer incidence,^15^ but this has been challenged.^29–31^ It has been suggested that recent exposure,^29–32^ initiating night work at young ages^16,30,31^ or before first pregnancy,^33,34^ and risk among pre-menopausal women for oestrogen receptor (ER) positive, progesterone receptor (PR) positive, or human epidermal growth factor-receptor 2 (HER2) positive tumours^14,16,34,35^ may be relevant, but the evidence is inconclusive. We therefore examined risk of breast cancer in relation to timing of night shift work and receptor status, in a large UK cohort study that has not been included in previous meta-analyses, using detailed questionnaire information at recruitment and during follow-up, with adjustment for potentially confounding factors.

## Methods

The Generations Study (GS) is a cohort study of >113,700 women aged 16 or older from the United Kingdom. Questionnaire information and informed consent was gained at recruitment since 2003.^36^ The first follow-up questionnaire was 2½ years after recruitment was completed by 99% of non-deceased participants, a second 6 years after recruitment by 97%, and a third 9½ years after recruitment by 96% of those recruited long enough ago to have entered this phase of follow-up.

Breast and other cancers occurring in the cohort were identified from recruitment and follow-up questionnaires, and spontaneous reports to the study centre. Spontaneous reports occurred when a woman contacted us and told us about her cancer diagnosis. For those lost to questionnaire follow-up, we ascertained cancers from linkage to National Health Service Central Registers (NHSCR), which provides information on vital status, cancer diagnosis and site.^37^ Confirmation of diagnosis was obtained from cancer registries in the United Kingdom, NHSCR linkage, pathology reports, and correspondence with patients’ general practitioners.

Information on risk factors for breast cancer was obtained from recruitment and follow-up questionnaires. Because we had collected ages or dates at which certain events or changes in lifestyle occurred, we were able to conduct analyses using time-updated alcohol use, parity, oral contraceptive use, menopausal hormone therapy (MHT) use, and menopausal status, at the ages these events or changes occurred through to the second follow-up questionnaire. We also updated post-menopausal body mass index (BMI) at the date of the second follow-up questionnaire.

In relation to night shift work, women were asked in the recruitment questionnaire: “Over the last ten years, have you had any jobs that regularly involved work in the late evening or night (between 10 pm and 7 am)”, and we collected information on type of job, year starting and ending, average number of nights per week working at night or late evening, and average number of hours worked between 10 pm and 7 am for each such episode of work. The same information on night work but covering the period from recruitment to the second follow-up questionnaire, was collected at this follow-up 6 years after recruitment. When analysing type of occupation in which night shift work occurred, if a woman reported different types of night work occupation concurrently during a time period, we counted only the type of work that she had done the most, so that we could allocate her to a single occupation at any one time. If the hours per night or nights per week of night work changed during a period of night work, we took an average of these night work intensity measures weighted by the number of years at each intensity. We did not ask about night shift work in the next follow-up questionnaire, 9½ years after recruitment.

To analyse breast cancer risk in relation to being a night shift worker in the last 10 years, we updated night shift work status, and cumulative duration and time since cessation in single year increments, through to the 6-year follow-up. After this point, we assumed that women who had never been a night shift worker, or had ceased, did not commence new night work, and that women who were in current night work continued at the same intensity, frequency, and duration through to the end of analytic follow-up. Because our questionnaires only solicited information on night shift work history which, at least in part, had been undertaken in the last 10 years, we did not count information from night shift histories that ended completely >10 years ago (i.e., when women volunteered more information than requested) because we deemed this would be incomplete or missing for some women. Therefore, we were able to analyse comprehensive information on night shift exposures more than ten years ago that continued into the last 10 year period, but for exposures based on work history that ended before this 10 year period our analysis would be less complete.

### Statistical analysis

The current analytic cohort is based on all women who were recruited to the study during June 2003–December 2014 without prior invasive or in-situ breast cancer or other malignancy except non-melanoma skin cancer, or prior mastectomy, and who did not report being registered as blind or partially sighted because of the possible association between blindness, melatonin, and breast cancer risk.^38^ The recruitment cut-off at December 2014 was selected because at the time of analysis the second follow-up was practically complete for this group of recruits and all women would have in principle been able to reach the minimum follow-up of 2½ years associated with the first follow-up questionnaire. Women entered risk at their date of recruitment and were censored at the earliest date of: invasive or in-situ breast cancer; other malignancy except non-melanoma skin cancer; death; or most recent follow-up questionnaire that was dependent on the date of recruitment. If the most recent follow-up questionnaire was not completed, the censoring date was the date the most recent follow-up questionnaire was due, if cancer and vital status were known after this date from NHSCR linkage. If cancer and vital status were not known at this due date, this was considered a loss to optimum follow-up and the censoring date was the date of the last completed questionnaire. The data for this analysis was extracted and frozen from our live database on 9-Jan-2019.

Left-truncated and right-censored Cox proportional hazards regression,^39^ using attained age as the implicit time scale, was used to estimate the hazard ratio (HR) and 95% confidence interval (95%CI) for night shift work and risk of first invasive breast cancer adjusted for potential confounding factors (see footnotes to Tables 2 and 3). We analysed primarily the risk in relation to invasive breast cancer so that our results would be comparable with those from the Million Women Study, EPIC-Oxford, and UK Biobank,^15^ the Nurses Health Studies,^16^ and other prospective studies,^9–11,15^ although we also compared HRs for invasive versus in-situ breast cancer.

Statistical trends for frequency, intensity, and duration of night work, and time since last night work, were based on discrete time-varying annually updated values. For example, if at the time of recruitment, a woman had been in night shift work for 5 years, her cumulative exposure would be 5 years. If she continued night shift work, then in the next year of follow-up, her cumulative exposure would be updated to 6 years, and so on as long as she continued night shift work. Trends in these time-varying exposures and trend in risk with age starting night work were assessed using the likelihood ratio test.^40^ For trend analyses, the groups defined by not being a night shift worker in the last 10 years and current night shift worker in analysis of time since cessation were not assigned a zero magnitude but were treated as separate categorical terms, as was the group where the details of night shift work were missing (i.e., trends were only evaluated across those who were exposed, but by using indicator variables, we included the non-exposed and missing value group in the regression analysis). We stratified by breast cancer risk factors to examine interactions between these risk factors and night shift work, and by ever-use of melatonin supplements (reported at cohort entry). Heterogeneity in HRs by sub-type of breast cancer defined by receptor status, histology, or invasive vs. in-situ breast cancer, was assessed using a data augmentation method^41^ and Wald test.^40^ All statistical tests were two-sided and analyses were conducted using Stata/IC version 14.2.^42^

## Results

During 2003–2014, a recruitment questionnaire was completed by 102,869 women in the GS who had no previous invasive or in-situ breast cancer or other malignancy except non-melanoma skin cancer. By the censoring date, 1.1% of the women had died. Of the remainder, cancer and vital status was known for 95.3% who had completed the relevant follow-up questionnaire, and a further 3.2% from linkage to the NHSCR. The remaining 0.4% were lost to the follow-up and censored at the date of an earlier returned questionnaire. The last follow-up and censoring date was 27th March 2018.

Table 1 presents descriptive characteristics, at recruitment, of the cohort members eligible for analysis, by night work status. The median age at recruitment was 45 years (inter-quartile range: 35–55). Among the participants, 17.5% reported being a night shift worker within the last 10 years. There were significant variations in the percentage of night shift workers in relation to all but one demographic or descriptive characteristic in the table (all *P*_heterogeneity_ < 0.0001, except for pre-menopausal oral contraceptive use, *P* = 0.13). The proportion reporting ever being a night shift worker in the 10 years before recruitment was greater for women who were younger, pre-menopausal, higher BMI at age 20, did not report family history of breast cancer or personal history of benign breast disease, lived in less affluent neighbourhoods, nulliparous, ex-drinkers of alcohol, current smokers, higher levels of physical activity, higher BMI at post-menopausal ages, current users of MHT, and those not reporting ever use of melatonin supplement.

**Table 1:** Characteristics at recruitment of 102,869 women from the Generations Study, by night shift work^a^ during the 10 years before recruitment to cohort

Characteristic	Ever been a night shift worker during the 10 years before recruitment to cohort					Total	
	No		Yes		Yes		
	*N*	*%*	*N*	*%*	*%* ^b^	*N*	*%*
*Year of birth*							
1908–1939	5771	*6.8*	136	*0.8*	*2.3*	5907	*5.7*
1940–1949	20174	*23.8*	1308	*7.3*	*6.1*	21482	*20.9*
1950–1959	21816	*25.7*	2930	*16.3*	*11.8*	24746	*24.1*
1960–1969	19066	*22.5*	4665	*25.9*	*19.7*	23731	*23.1*
1970–1998	18061	*21.3*	8942	*49.7*	*33.1*	27003	*26.2*
*Year of recruitment*							
2003–2005	27481	*32.4*	6272	*34.9*	*18.6*	33753	*32.8*
2006­–2007	40096	*47.2*	8293	*46.1*	*17.1*	48389	*47.0*
2008–2014	17311	*20.4*	3416	*19.0*	*16.5*	20727	*20.1*
*Age at recruitment (years)*							
16–34	15142	*17.8*	8037	*44.7*	*34.7*	23179	*22.5*
35–44	18295	*21.6*	4807	*26.7*	*20.8*	23102	*22.5*
45–54	21112	*24.9*	3281	*18.2*	*13.4*	24393	*23.7*
55–64	22277	*26.2*	1602	*8.9*	*6.7*	23879	*23.2*
65–74	7088	*8.3*	244	*1.4*	*3.3*	7332	*7.1*
75–102	974	*1.1*	10	*0.1*	*1.0*	984	*1.0*
*BMI at recruitment (kg/m* ^*2*^ *)*							
<20.0	5398	*6.4*	1278	*7.1*	*19.1*	6676	*6.5*
20.0– <22.5	18074	*21.3*	3897	*21.7*	*17.7*	21971	*21.4*
22.5– <25.0	23022	*27.1*	4457	*24.8*	*16.2*	27479	*26.7*
25.0– <30.0	24817	*29.2*	4832	*26.9*	*16.3*	29649	*28.8*
≥30.0	11177	*13.2*	2844	*15.8*	*20.3*	14021	*13.6*
Missing	2400	*2.8*	673	*3.7*	*21.9*	3073	*3.0*
*BMI at age 20* *(kg/m* ^*2*^ *)*							
<18.5	7075	*8.3*	1198	*6.7*	*14.5*	8273	*8.0*
18.5– <20.0	16275	*19.2*	2822	*15.7*	*14.8*	19097	*18.6*
20.0– <22.5	32370	*38.1*	6219	*34.6*	*16.1*	38589	*37.5*
22.5– <25.0	17435	*20.5*	4200	*23.4*	*19.4*	21635	*21.0*
≥25.0	7817	*9.2*	2807	*15.6*	*26.4*	10624	*10.3*
age <20 at entry	982	*1.2*	220	*1.2*	*18.3*	1202	*1.2*
Missing	2934	*3.5*	515	*2.9*	*14.9*	3449	*3.4*
*Family history of breast cancer at recruitment*							
None reported	71348	*84.0*	15592	*86.7*	*17.9*	86940	*84.5*
Yes	13540	*16.0*	2389	*13.3*	*15.0*	15929	*15.5*
*History of benign breast disease at recruitment*							
None reported	68014	*80.1*	15033	*83.6*	*18.1*	83047	*80.7*
Yes	16874	*19.9*	2948	*16.4*	*14.9*	19822	*19.3*
*Living in affluent neighbourhood* ^c^							
More affluent	38913	*45.8*	5723	*31.8*	*12.8*	44636	*43.4*
Less affluent	45987	*54.2*	12258	*68.2*	*21.0*	58233	*56.6*
*Parity at recruitment*							
Nulliparous	20798	*24.5*	8001	*44.5*	*27.8*	28799	*28.0*
Parous	64090	*75.5*	9980	*55.5*	*13.5*	74070	*72.0*
*Premenopausal oral contraceptive use*							
Post-menopausal^d^	39789	–	3251	–	–	43040	–
No	6364	*14.1*	2006	*13.6*	*24.0*	8370	*14.0*
Yes	38735	*85.9*	12724	*86.4*	*24.7*	51459	*86.0*
*Regular alcohol consumption*							
Never	16928	*19.9*	3204	*17.8*	*15.9*	20132	*19.6*
Current	57364	*67.6*	11959	*66.5*	*17.3*	69323	*67.4*
Ex-drinker	10596	*12.5*	2818	*15.7*	*21.0*	13414	*13.0*
*Regular cigarette smoking*							
Never	55236	*65.1*	10740	*59.7*	*16.3*	65976	*64.1*
Current	5847	*6.9*	2544	*14.1*	*30.3*	8391	*8.2*
Ex-smoker	23805	*28.0*	4697	*26.1*	*16.5*	28502	*27.7*
*Physical activity (by quartile of MET)*							
<30	21913	*25.8*	3871	*21.5*	*15.0*	25784	*25.1*
30– <51	21726	*25.6*	4145	*23.1*	*16.0*	25871	*25.1*
51– <83	21044	*24.8*	4275	*23.8*	*16.9*	25319	*24.6*
83+	20064	*23.6*	5669	*31.5*	*22.0*	25733	*25.0*
Missing	141	*0.2*	21	*0.1*	*13.0*	162	*0.2*
*Post-menopausal BMI (kg/m* ^*2*^ *)*							
Pre-menopausal^d^	45099	–	14730	–	–	59829	–
<20.0	1511	*3.8*	96	*3.0*	*6.0*	1607	*3.7*
20.0– <22.5	6481	*16.3*	450	*13.8*	*6.5*	6931	*16.1*
22.5– <25.0	10715	*26.9*	769	*23.7*	*6.7*	11484	*26.7*
25.0– <30.0	13244	*33.3*	1092	*33.6*	*7.6*	14336	*33.3*
30.0+	5482	*13.8*	677	*20.8*	*11.0*	6159	*14.3*
Missing	2356	*5.9*	167	*5.1*	*6.6*	2523	*5.9*
*Post-menopausal hormone therapy use*							
Pre-menopausal^d^	45099	–	14730	–	–	59829	–
Never used	22696	*57.0*	1795	*55.2*	*7.3*	24491	*56.9*
Ex-user	9944	*25.0*	707	*21.7*	*6.6*	10651	*24.7*
Current user	6324	*15.9*	675	*20.8*	*9.6*	6999	*16.3*
User, status unknown	825	*2.1*	74	*2.3*	*8.2*	899	*2.1*
*Ever used melatonin supplements before recruitment*							
No	82010	*96.6*	17487	*97.3*	*17.6*	99497	*96.7*
Yes	2878	*3.4*	494	*2.7*	*14.7*	3372	*3.3*
Total	84888	*100.0*	17981	*100.0*	*17.5*	102869	*100.0*

*BMI* body mass index, *MET* metabolic equivalent to task

^a^Night shift work: Over the last ten years, have you had any jobs that regularly involved work in the late evening or night (between 10 pm and 7am)?

^b^Row percentage

^c^Based on ACORN score, a socio-demographic neighbourhood based score

^d^Not included in column percentage for distribution

During 880,864 person-years (median 9.5 years) of follow-up 2059 invasive breast cancers occurred, of which 2041 were confirmed through national cancer registration or medical records, and the remainder (*n* = 18) were self-reported mostly with treatment or other details that implied breast cancer, e.g., information on receptor status. ER status data were available for 96.6%, and of these, 84.3% were ER-positive. Invasive ductal carcinoma accounted for 78.2%, and lobular for 16.4%, of tumours. Further descriptive characteristics of the breast cancer cases are given in Supplementary Table 1.

The HR for invasive breast cancer in relation to being a night shift worker within the last 10 years was 0.98 (95%CI: 0.85–1.14; *P* = 0.80) with adjustment only for attained age, and 1.00 (95%CI: 0.86–1.15; *P* = 0.96) with adjustment for additional potentially confounding factors (Table 2). There were no significantly raised risks in relation to hours worked per night (*P*_trend_ = 0.62), nights per week on night shift (*P*_trend_ = 0.066), cumulative years of employment as a night shift worker (*P*_*trend*_ = 0.51), or cumulative hours of night shift work (*P*_trend_ = 0.51), but there was a significant positive trend with average hours per week, adjusted for age only (*P*_trend_ = 0.038) or fully adjusted (*P*_trend_ = 0.035) (Table 2).

**Table 2:** Relative risk of invasive breast cancer in relation to ever being a night shift worker^a^ in last 10 years, by frequency, intensity, and duration of night shift work

	Cases^b^	Person-years (per 100,000)	Age adjusted			Full adjustment^c^		
			HR	95% CI	*P*-value	HR	95% CI	*P*-value
*Being a night shift worker within the last 10 years*								
None	1845	738.4	1.00	Baseline		1.00	Baseline	
Yes	214	142.5	0.98	0.85–1.14	0.80	1.00	0.86–1.15	0.96
*Average hours worked per night*								
None	1845	738.4	1.00	Baseline		1.00	Baseline	
<7 h	91	66.4	1.05	0.85–1.30	0.66	1.04	0.84–1.28	0.74
7+hours	103	65.1	0.93	0.76–1.14	0.48	0.96	0.78–1.17	0.68
Unknown	20	11.0	0.98	0.63–1.52	0.93	1.02	0.65–1.58	0.94
Trend^d^:					0.44			0.62
*Average nights per week on night shift*								
None	1845	738.4	1.00	Baseline		1.00	Baseline	
<4	152	104.9	0.95	0.80–1.12	0.52	0.96	0.81–1.14	0.65
4–7	55	32.5	1.16	0.89–1.52	0.28	1.18	0.90–1.55	0.23
Unknown	7	5.1	0.70	0.33–1.48	0.35	0.70	0.33–1.47	0.35
Trend^d^:					0.073			0.066
*Average hours per week on night shift*								
None	1845	738.4	1.00	Baseline		1.00	Baseline	
<10	70	56.3	0.88	0.69–1.12	0.31	0.88	0.69–1.12	0.29
10– <20	61	38.9	1.06	0.82–1.37	0.67	1.07	0.83–1.39	0.60
20– <30	35	20.5	1.02	0.73–1.43	0.90	1.05	0.75–1.48	0.76
30+	26	13.5	1.20	0.81–1.77	0.36	1.27	0.86–1.87	0.24
Unknown	22	13.3	0.88	0.58–1.34	0.54	0.91	0.60–1.38	0.65
Trend^d^:					0.038			0.035
*Cumulative years of employment as night shift worker*								
None	1845	738.4	1.00	Baseline		1.00	Baseline	
<10	89	87.0	0.90	0.73–1.12	0.36	0.92	0.74–1.14	0.44
10– <20	65	33.7	1.07	0.83–1.37	0.61	1.09	0.85–1.40	0.51
20– <30	36	14.9	0.96	0.69–1.34	0.81	0.97	0.70–1.35	0.85
30+	24	6.9	1.12	0.75–1.67	0.59	1.12	0.75–1.69	0.57
Trend^d^:					0.49			0.51
*Cumulative hours of night shift work (10,000* *h)*								
None	1845	738.5	1.00	Baseline		1.00	Baseline	
0– <1	103	83.0	1.00	0.82–1.23	0.99	1.00	0.82–1.23	0.98
1– <2	36	18.7	1.01	0.73–1.41	0.95	1.04	0.74–1.44	0.83
2– <3	22	8.1	1.21	0.80–1.85	0.37	1.24	0.82–1.90	0.31
3+	21	7.4	1.05	0.68–1.61	0.84	1.07	0.70–1.66	0.75
Unknown	32	25.3	0.78	0.55–1.10	0.16	0.79	0.56–1.13	0.20
Trend^d^					0.57			0.51

*HR* hazard ratio, *CI* confidence interval

^a^Night shift work: Over the last ten years, have you had any jobs that regularly involved work in the late evening or night (between 10 pm and 7am)?

^b^Number of breast cancer cases

^c^HR adjusted for: attained age (Cox regression time scale); time since recruitment to cohort (0, 1–2, 3 +years); birth cohort (1908–39, 1940–49, 1950–59, 1960–69, 1970–96); benign breast disease (yes, no); family history of breast cancer in 1st degree relatives (yes, no); socio-economic score (ACORN score as trend, missing); birth weight (trend, missing); height at age 20 (trend, missing); age at menarche (trend, missing); body mass index at age 20 (trend, missing); age at first pregnancy (trend, missing); parity (trend, missing); breast-feeding (yes/no); current oral contraceptive use before menopause (yes, no); alcohol consumption (never regular, trend current drinker 1­– <60 g/day, current drinker 60 +g/day, past drinker, drinker with unknown details); age started smoking (never, < 17, 17_9, 20+, unknown); physical activity (log(metabolic equivalent) trend, missing); post-menopausal body mass index (trend, missing); menopausal hormone therapy use (never used, ex-user, current oestrogen only user, current oestrogen plus progestogen user, current user of other types, missing); menopausal status (pre- or post-menopausal) and age at menopause (trend, missing)

^d^Trend evaluated over those doing night shift work, based on time-varying annually updated values

There were no significantly raised risks with being a night shift worker in the last 10 years by type of occupation nor was there evidence for heterogeneity (Table 3; *P* = 0.20). There were no significant associations with age started night shift work (*P*_trend_ = 0.89), whether night shift work started before (*P* = 0.73) or after (*P* = 0.90) first pregnancy, or by time since last worked night shifts (*P*_trend_ = 0.38). When we restricted our analyses to women at recruitment who either reported being in a paid or self-employed job (*n* = 69,942), student (*n* = 3599), unemployed (*n* = 789), or retired (*n* = 15,711) our results were essentially the same (Supplementary Tables 2 and 3). We examined by stratification, interactions between risk of breast cancer and night shift work by selected risk factors for breast cancer, and for ever-use of melatonin supplement before recruitment but found no significant associations or interactions (Supplementary Table 4).

**Table 3:** Relative risk of invasive breast cancer in relation to ever being a night shift worker^a^ in last 10 years, by type of work, age started, timing of first pregnancy, and time since last night shift work

	Cases^b^	Person-years (per 100,000)	Age adjusted			Full adjustment^c^		
			HR	95% CI	*P*-value	HR	95% CI	*P*-value
*Type of night shift work in last 10 years*								
None	1845	763.4	1.00	Baseline		1.00	Baseline	
Nurse	83	51.1	0.83	0.66–1.03	0.092	0.85	0.68–1.07	0.17
Waitress	10	24.5	0.60	0.32–1.13	0.11	0.61	0.33–1.15	0.13
Office/shops	29	14.3	1.32	0.92–1.91	0.14	1.35	0.93–1.95	0.11
Health Carer	22	9.1	1.29	0.85–1.97	0.23	1.42	0.93–2.17	0.11
Technical	18	8.4	1.37	0.86–2.18	0.18	1.28	0.81–2.05	0.29
Emergency services, armed forces, air crew	13	7.0	1.37	0.79–2.37	0.26	1.31	0.76–2.27	0.34
Manual work	8	4.6	1.10	0.55–2.21	0.79	1.12	0.56–2.25	0.74
Doctor/GP	7	8.3	0.85	0.40–1.78	0.66	0.78	0.37–1.65	0.52
Other	24	20.7	0.99	0.66–1.49	0.98	0.97	0.65–1.45	0.88
Heterogeneity:					0.18			0.20
*Age started night work (years)*								
None	1845	738.4	1.00	Baseline		1.00	Baseline	
<25	71	72.7	1.02	0.80–1.31	0.86	1.03	0.80–1.32	0.82
25–34	45	36.1	0.82	0.61–1.11	0.20	0.84	0.62–1.14	0.26
35–44	63	20.7	1.21	0.94–1.56	0.13	1.24	0.96–1.60	0.095
45+	35	13.0	0.84	0.60–1.17	0.30	0.84	0.60–1.18	0.32
Trend:					0.86			0.89
*Night work in relation to first pregnancy, parous women only*								
Parous but no night work	1593	586.5	1.00	Baseline		1.00	Baseline	
Started night work before 1^st^ pregnancy	58	41.3	1.00	0.77–1.31	0.99	0.95	0.73–1.25	0.73
Started night work after 1^st^ pregnancy	111	47.0	0.97	0.80–1.18	0.75	1.01	0.83–1.23	0.90
*Time since last night shift work (years)*								
None	1845	738.4	1.00	Baseline		1.00	Baseline	
Current	84	54.7	0.98	0.79–1.23	0.88	1.01	0.80–1.26	0.96
0– <5	60	40.5	1.05	0.81–1.36	0.72	1.05	0.81–1.36	0.72
5– <10	70	47.3	0.93	0.73–1.18	0.55	0.94	0.74–1.20	0.64
Trend^d^:					0.34			0.38

*HR* hazard ratio, *CI* confidence interval

^a^Night shift work: Over the last ten years, have you had any jobs that regularly involved work in the late evening or night (between 10 pm and 7am)?

^b^Number of breast cancer cases

^c^HR adjusted for: attained age (Cox regression time scale); time since recruitment to cohort (0, 1–2, 3 +years); birth cohort (1908–39, 1940–49, 1950–59, 1960–69, 1970–96); benign breast disease (yes, no); family history of breast cancer in 1st degree relatives (yes, no); socio-economic score (ACORN score as trend, missing); birth weight (trend, missing); height at age 20 (trend, missing); age at menarche (trend, missing); body mass index at age 20 (trend, missing); age at first pregnancy (trend, missing); parity (trend, missing); breast-feeding (yes/no); current oral contraceptive use before menopause (yes, no); alcohol consumption (never regular, trend current drinker 1­– < 60 g/day, current drinker 60 +g/day, past drinker, drinker with unknown details); age started smoking (never, <17, 17_9, 20+, unknown); physical activity (log(metabolic equivalent) trend, missing); post-menopausal body mass index (trend, missing); menopausal hormone therapy use (never used, ex-user, current oestrogen only user, current oestrogen plus progestogen user, current user of other types, missing); menopausal status (pre- or post-menopausal) and age at menopause (trend, missing)

^d^Trend evaluated over those who have ceased night shift work, based on time-varying annually updated values

We also stratified by menopausal status and examined risk in relation to being a night shift worker in the last 10 years and average hours per week on night shift but found no significant associations, trends, or interactions (Supplementary Table 5). Nor were there significantly raised risks in relation to night shift work by receptor status of breast cancer (ER, PR, HER2) or histological type (Supplementary Table 5). Further sub-division by menopausal status and sub-type of breast cancer is shown in Supplementary Table 6. No significantly raised risks were seen by sub-type for pre-menopausal women, although there were some significant associations and trends seen in post-menopausal women for PR-positive, HER2-negative, and lobular tumours, but with no consistent pattern. Finally, when we analysed risk in relation to in-situ breast cancer (*n* = 411 cases) the adjusted HR for night shift work in the last 10 years was 1.16 (95%CI: 0.85–1.57; *P* = 0.35), with no significant heterogeneity between invasive and in-situ breast cancer (*P* = 0.39).

## Discussion

In our detailed analysis of night shift work and risk of breast cancer, we examined relative risks in relation to a number of aspects of being a night shift worker in the last 10 years, and with few exceptions, most were not statistically significant. We found no evidence for an overall increase in risk of breast cancer for women who had been night shift workers within the last 10 years, or by hours worked per night, nights worked per week, average hours worked per week, cumulative years of employment, cumulative hours, or time since cessation of such work. We found no significantly raised risks with type of night shift occupation, by age at starting night shift work, or by age starting in relation to first pregnancy. Increased risk has been reported previously for night shift work specifically among nurses,^17^ in particular those engaged in rotating night shift work,^6,7,43^ but we found no increased risk for nurses undertaking night shift work, nor did the Million Women Study,^15^ although neither we nor the Million Women Study explicitly examined rotating shift work.

The Nurses’ Health Studies reported raised risks for night work of >20 years duration,^6,7^ but this has not been seen in other cohorts^9,11–13,15^ or the recent comprehensive meta-analysis of prospective studies.^15^ A previous dose–response meta-analysis of cohort and case-control studies did report a significant trend with duration^19^ whereas another did not,^17^ so the literature is inconclusive on this. We found no association with duration in our study, although our data for long durations of night work was limited because our study was focussed on recent night shift work in the last 10 years. One potential interpretation for the raised risk seen with long duration in the Nurses’ Health Studies is that this raised risk relates to night shift work during the period after puberty and before first childbirth, when the breast may be particularly susceptible to adverse changes.^16^ Such an association has been reported in a case-control study from France^33^ but we, and the Nurses’ Health Study-II,^16^ did not find increased risks with night shift work starting before first pregnancy. We also found no association with age started night shift work, similar to the one other cohort that has reported on this.^9^

We did not find evidence for significant interaction (effect modification) between breast cancer risk factors and night shift work in relation to risk of breast cancer, similar to the Million Women Study.^15^ However, we found night shift work was positively associated with several characteristics that are associated with breast cancer risk therefore there is scope for confounding. In our study adjusting for these potential confounding factors made no material changes to the results, and adjustment for similar factors in other cohort studies^6,7,9,10,12,14–16^ generally made little difference to their conclusions.^44^ But if the confounding association is study specific, or women who engage in night shift work differ in other unidentified ways from those who do not, this may explain why results in the literature have been inconsistent.

The only statistically significant association observed in our main analyses in relation to breast cancer and night shift work was a dose–response trend with average hours per week on night shift. A significant trend has previously been reported in a case-control study for hours per week of ‘graveyard’ shift work (*i.e*. work between 7 pm and 9 am) in the 10 years before diagnosis,^32^ and raised risk for ≥20 h per week in a pooled case-control analysis,^22^ whereas in a large record linkage cohort study based on Dutch Labor Force Surveys no significant association was seen with a metric based on contractual night working hours.^11^ There does not appear to be a strong rationale for hours per week on night shift being a risk factor for breast cancer. We found no significant overall association of risk of breast cancer with being a night shift worker. There was also lack of association with the other measures of dose, duration, and intensity that we analysed. These results from our study and the absence in the literature of an hypothesis for why there should be an association with hours per week but not with other measures of dose, duration, or intensity, and conflicting results from other studies,^11,22,32^ suggesting that it is possible that this is a chance finding in our data. However, unlike traditional carcinogens where cumulative exposure and dose may supersede intensity as a requisite for cause-effect association, it is still unclear which exposure ‘domains’ may be important in relation to night shift work.^28^

We found overall no significantly raised risks by menopausal status for ER, PR, HER2, or histological sub-types of breast cancer. Significantly raised risks have been reported for ER-positive,^16,34,35,45^ ER-negative,^46^ PR-positive,^16,34,35,45^ HER2-positive,^35^ HER2-negative,^45^ and lobular^34^ breast cancer sub-types in particular among pre-menopausal women^14,16,34,35^ but we did not find any significantly raised risks in pre-menopausal women, overall or by sub-type. We did see raised risks among post-menopausal women but are uncertain about the interpretation because these occurred in sub-group analyses subject to inflated type-I statistical error. The evidence in the literature for risk by tumour sub-type, and menopausal status, is inconsistent^14,16,34,35,45,46^ and this may be a reflection of small number of cases in subgroups and lack of statistical power, or that there is no substantive difference by sub-type of breast cancer in relation to night shift work.

Our night shift information was gained at recruitment and from follow-up questionnaire 6 years later, but our follow-up for breast cancer extended beyond the 6-year questionnaire. We updated night shift work up to the 6-year questionnaire, and then carried forward the exposure status at that point in time until end of follow-up if this was >6 years. About one-third (31%) of accrued person-years were after the 6-year follow-up, but 78% of the post 6-year follow-up person-years occurred soon after that, within the first three years after that follow-up. So the scope for potential exposure misclassification was therefore less than in cohort studies that implicitly carry forward exposure status from recruitment for everyone because the studies do not have any updated exposure information. The only other study with updated night shift exposure information using repeat questionnaires, The Nurses’ Health Study-II,^16^ found a significantly raised risk with cumulative duration ≥20 years, but we did not and other cohorts with updated night shift information based on record linkage to employment databases did not find an association overall or with duration.^8,13,14^

Our questions on night shift work only ascertained periods of shift work that took place, or at least ended, during the 10 years before recruitment. We did not collect lifetime history of night shift work because we needed to contain the burden of data collection on the recruits to the study, who at recruitment were completing a 44-page questionnaire covering a wide range of breast cancer related topics. Some have suggested that any increased risk associated with night shift work may diminish soon after exposure ceases^22,30,31^ and as analysed here recent, rather than historic, night shift work may be the most appropriate measure of exposure. However, to the extent that it is longer term or earlier exposures that matter, our analyses would be limited and weaker.

An advantage of our study is that we were able to examine a wider range of night shift exposures than most other prospective studies, which were often limited to analyses of duration of night shift. There was little scope for bias from unascertained mortality or exits, or erroneous reporting of breast cancer in our study, because follow-up for vital and breast cancer status was obtained for 99% of participants, and confirmation of reported breast cancers for over 99%. Our breast cancer cases were particularly well characterised for histological type and ER status, allowing for analyses by sub-type of breast cancer. Information on PR and HER2 status was less complete because these tests have not been conducted routinely throughout the study period. As breast cancer treatment has advanced, PR testing has become less common and HER2 testing become more common in the UK, but it seems unlikely this would cause major bias in our analyses by sub-type.

The interest in night shift work and risk of breast cancer springs from the hypothesis that exposure to light at night may increase risk of breast cancer.^1–3^ Only this GS cohort^47^ and a cohort of California teachers^48^ have examined directly, by prospective questionnaire, exposure to indoor light at night in relation to breast cancer risk, rather than outdoor light at night from ecological geographical correlation with environmental data (e.g., from satellite imagery), and both studies fail to find significant associations with indoor light at night. However, the possible association between night shift work and breast cancer remains a public health concern. A substantial proportion of women in the general population are exposed to night shift work^28,49^ and even a modestly increased risk could lead to considerable numbers of breast cancer cases^49–51^ if there were a causal association. We did find a statistically significant trend with average hours per week on night shift. But in the absence of an association between breast cancer risk and night shift work overall, or by other measures of dose, duration, or intensity, in our study, and no evidence for association from the most recent and comprehensive meta-analysis (not including the Generations Study) of prospective studies,^15^ our finding of a significant trend on its own does not provide strong support for a real causal association. Our data overall do not provide evidence for an increased risk of breast cancer with night shift work.

## Supplementary information

Supplementary Table 1

Supplementary Table 2

Supplementary Table 3

Supplementary Table 4

Supplementary Table 5

Supplementary Table 6

## Data Availability

The datasets generated during and/or analysed during the current study are not publicly available due to confidentiality reasons, but anonymised versions may be available from the corresponding author on reasonable request.
